# Assessing demographic and economic vulnerabilities to sea level rise in Bangladesh via a nighttime light-based cellular automata model

**DOI:** 10.1038/s41598-023-40329-9

**Published:** 2023-08-16

**Authors:** Bijoy Mitra, Syed Masiur Rahman, Mohammed Sakib Uddin, Khaled Mahmud, Md Kamrul Islam, Md Arifuzzaman, MM Hafizur Rahman, Muhammad Muhitur Rahman

**Affiliations:** 1 Department of Geography and Environmental Studies, University of Chittagong, Chittagong, 4331 Bangladesh; 2 Applied Research Center for Environment and Marine Studies, Research Institute, King Fahd University of Petroleum and Minerals, 31261 Dhahran, Saudi Arabia; 3 Department of Civil and Environmental Engineering, College of Engineering, King Faisal University, 31982 Al-Ahsa, Saudi Arabia; 4 Department of Communication and Networking, College of Computer and Information Sciences, King Faisal University, 31982 Al-Ahsa, Saudi Arabia

**Keywords:** Climate change, Computer science

## Abstract

The Intergovernmental Panel on Climate Change (IPCC) 6th Assessment Report (AR6) forecasts a sea level rise (SLR) of up to 2 m by 2100, which poses significant risks to regional geomorphology. As a country with a rapidly developing economy and substantial population, Bangladesh confronts unique challenges due to its extensive floodplains and 720 km-long Bay of Bengal coastline. This study uses nighttime light data to investigate the demographic repercussions and potential disruptions to economic clusters arising from land inundation attributable to SLR in the Bay of Bengal. By using geographical information system (GIS)-based bathtub modeling, this research scrutinizes potential risk zones under three selected shared socioeconomic pathway (SSP) scenarios. The analysis anticipates that between 0.8 and 2.8 thousand km^2^ of land may be inundated according to the present elevation profile, affecting 0.5–2.8 million people in Bangladesh by 2150. Moreover, artificial neural network (ANN)-based cellular automata modeling is used to determine economic clusters at risk from SLR impacts. These findings emphasize the urgency for land planners to incorporate modeling and sea inundation projections to tackle the inherent uncertainty in SLR estimations and devise effective coastal flooding mitigation strategies. This study provides valuable insights for policy development and long-term planning in coastal regions, especially for areas with a limited availability of relevant data.

## Introduction

Sea level rise (SLR) is the change in the height of the ocean surface in response to climatic changes. Principally, the mean sea level rises by two mechanisms: first, melting ice sheets and glaciers, which create runoff that enters the ocean. Second, because warm water takes up more space than cold water, the volume of water in the ocean increases^1,2^. The IPCC has outlined a variety of natural and anthropogenic perspectives to describe the trends in global mean sea level (GMSL). In 2021, the IPCC published its 6th assessment report (AR6), which presented an estimation and likely fluctuations of mean sea level at local and global magnitudes. The IPCC approximates the GMSL rise and its impact by using semiempirical predictions based on a few scenarios highlighted in their IPCC assessment reports^2–5^. In AR6, the IPCC mentions 5 SSPs (shared socioeconomic pathways) by evaluating how demographic changes, economic growth, and technological advancement may affect greenhouse gas emissions and climate change in various socioeconomic situations. For instance, SSP1–1.9 is viewed as the lowest emissions scenario in which the global mean temperature is limited to 1–1.8 °C by 2100 with net zero carbon emissions, rigorous climatic policy, and sustainable development. In contrast, SSP5–8.5 is considered the high emission scenario, with weaker climatic regulations and a predicted mean temperature rise of 3.3–5.7 °C by 2100 compared to preindustrial levels^5–7^.

SLR has now emerged as a significant cause for concern, as the mean sea level continues to rise. The IPCC predicted that the GMSL would rise to 0.52–0.98 m in the 5th Assessment Report^3^; however, this prediction has been increased to 2 m by 2100, as published in AR6^2,5^. Furthermore, Wang et al.^8^ measured the GMSL rise via satellite instruments (e.g., 0.053 ± 0.026 mm year^−2^ from the Goddard Space Flight Center, National Aeronautics and Space Administration (NASA)); the results correspond to the SSP2–4.5 and SSP5–8.5 projections. Moreover, on the regional scale, Wang et al.^8^ measured several tide-gauge records, and their weighted-average statistics (0.063 ± 0.120 mm year^−2^) also validate the SSP2–4.5 and SSP5–8.5 projections. By analyzing future SLR in the China Sea, Qu et al.^9^ projected that the SLR would be 48–61 cm under SSP2–4.5 and 84–99 cm under the high-end SSP5–8.5 scenario.

The Bay of Bengal (BOB), the northwestern arm of the Indian Ocean, is a partially enclosed basin that receives significant monsoon winds and frequent reversing circulation. It has a complex tropical coastal ecosystem, extensive river deposition in the bay's northern part, and copious wetlands, marshes, and mangroves associated with its land areas^10–12^. The bay incorporates the world's largest mangrove forest (Sundarbans) and constitutes one of the most significant blue economic zones for its inhabitants. However, the enormous rivers of the Indian subcontinent contribute significantly to the bay's water properties and stratification by releasing vast volumes of fresh water in the northern portion^13^. Therefore, at higher altitudes, the BOB exhibits greater sea level variation, whereas the mid altitude of the BOB and the northern Arabian Sea demonstrate relatively low sea surface height variability^10,14^.

Sea level changes are also influenced by the current decline in glaciers and shifts in land water storage, together with variations in the gravitational pull of the Earth and lateral land movement. For instance, recent studies have demonstrated the effect of excessive groundwater dependencies leading to the threat of subsidence in low-lying deltaic plains^15,16^. In Bangladesh, subsidence can be divided into two significant causalities^17^. One is associated with active tectonic forces and sediment loads due to Himalayan upwelling, and the other is attributed to the dryness and flattening of the Proto-Bengal Fan shale and mud^18^. Furthermore, the rate of sedimentation in Ganges–Brahmaputra–Meghna has significantly decreased by 10 MT/year, further enhancing the potential subsidence of the corresponding riverbanks. Consequently, a 0.6–5.5 mm/year subsidence has been observed in the Surma River, while a 1–2 mm/year subsidence has been estimated in the Ganges deltaic at Calcutta, Khulna, and the Sundarbans^17^.

The prediction of coastal mean sea level and extreme sea levels informs coastal impact and hazard identification, synchronization initiatives, and protracted decision-making. It also illustrates how SLR will affect people's adaptability and the origins and hotspots of potential migration. However, in comparison to in situ observations, recent developments in satellite altimetry enable very accurate estimates of sea surface height with excellent spatial and temporal precision. Surface circulation and mesoscale phenomena such as fronts, eddies, and vertical motions have been created using satellite altimetry-derived sea surface height data. Moreover, to project SLR on a local or regional scale, the IPCC introduced inundation scenarios to identify significant changes correlating with coastal elevations. However, several methodologies have also been established to assess future projections. In the mid-1980s, to detect climate change near the coast of the US, “The Sea Level Affecting Marshes Model” (SLAMM) was developed^19^. Then, in the 1990s, Costanza et al.^20^ demonstrated the “Ecological Landscape Spatial Simulation Model” to predict climatic changes by observing environmental variables^20,21^. A few software-based models, e.g., the Dynamic Interactive Vulnerability Assessment (DIVA) and SimCLIM, have also been developed to predict SLR at the local and regional levels^22,23^. At present, numerous researchers utilize geographic information systems (GIS) and geospatial modeling tools to examine the impacts of SLR on coastal ecosystems^24–26^. GIS and geospatial research methods are significant for determining the influence of SLR at various spatial and temporal scales.

Our research aims to address a significant gap in the literature regarding the effects of SLR in low-elevation nations by using global threshold data and models to evaluate migration scenarios influenced by climatic changes. A GIS technique-based bathtub model that considers three SLR scenarios is used to demonstrate the possible coastal inundation due to SLR in the BOB. To project the prospective SLR, the IPCC's SLR data for the SSP1–1.9, SSP2–4.5, and SSP5–8.5 scenarios until 2150 were combined. Hence, our findings are significantly reliant on future extreme climate consequences. The bathtub approach indicates all the inundated areas below a user-specified elevation, identical to that of a water container or single-value water surface^27^. This model is simple to implement and able to evaluate the potential global coastal flood risk^28,29^. The method, however, occasionally fails to estimate the magnitude of flooding since it does not account for sea level amplification based on estuarine morphology^30^. In contrast, our study encompasses the entirety of Bangladesh as a research area to quantify the projected inundation resulting from SLR pertaining to GMSL rise. However, it is also paramount to predict the possible threat to anthropogenic infrastructures due to SLR. Therefore, day/night band (DNB) composite nighttime light data extracted from the Visible Infrared Imaging Radiometer Suite (VIIRS) are used to evaluate economically significant clusters in Bangladesh. The use of nighttime light data as a substitution for economic activity is widely accepted in remote sensing and other specializations^31,32^. Furthermore, the Modules for Land-Use Change Simulation (MOLUSCE) simulation tool was used to model the future economic clusters for 2050, 2100, and 2150 using the DNB composite raster of Bangladesh^33,34^. Based on the multilayer perceptron artificial neural network (MP-ANN) technique, the MOLUSCE plugin uses cellular automata statistics to simulate potential land-use decisions based on preceding consequences. Here, artificial neural network (ANN) algorithms were used to train the DNB composite raster from 2014 to 2022 to simulate the transitional prospects of economic clusters. The prospective inundated area was then characterized in the simulated models in relation to space and time utilizing ArcMap 10.8.

This study presents a novel approach to assessing demographic and economic vulnerabilities arising from SLR in Bangladesh, a country challenged by data scarcity. By integrating nighttime light data as a proxy for population and economic activities with GIS-based bathtub modeling and ANN-based MOLUSCE modeling, this research investigates potential risk zones under various SSP scenarios. The methodology offers a detailed evaluation of future demographic impacts and disruptions to economic clusters, thus providing valuable guidance for effective policy-making and long-term coastal planning in Bangladesh and demonstrating the potential of nighttime light data in addressing data scarcity issues in similar regions.

## Result analysis

### Sea level projection under different scenarios

**Figure 1:**
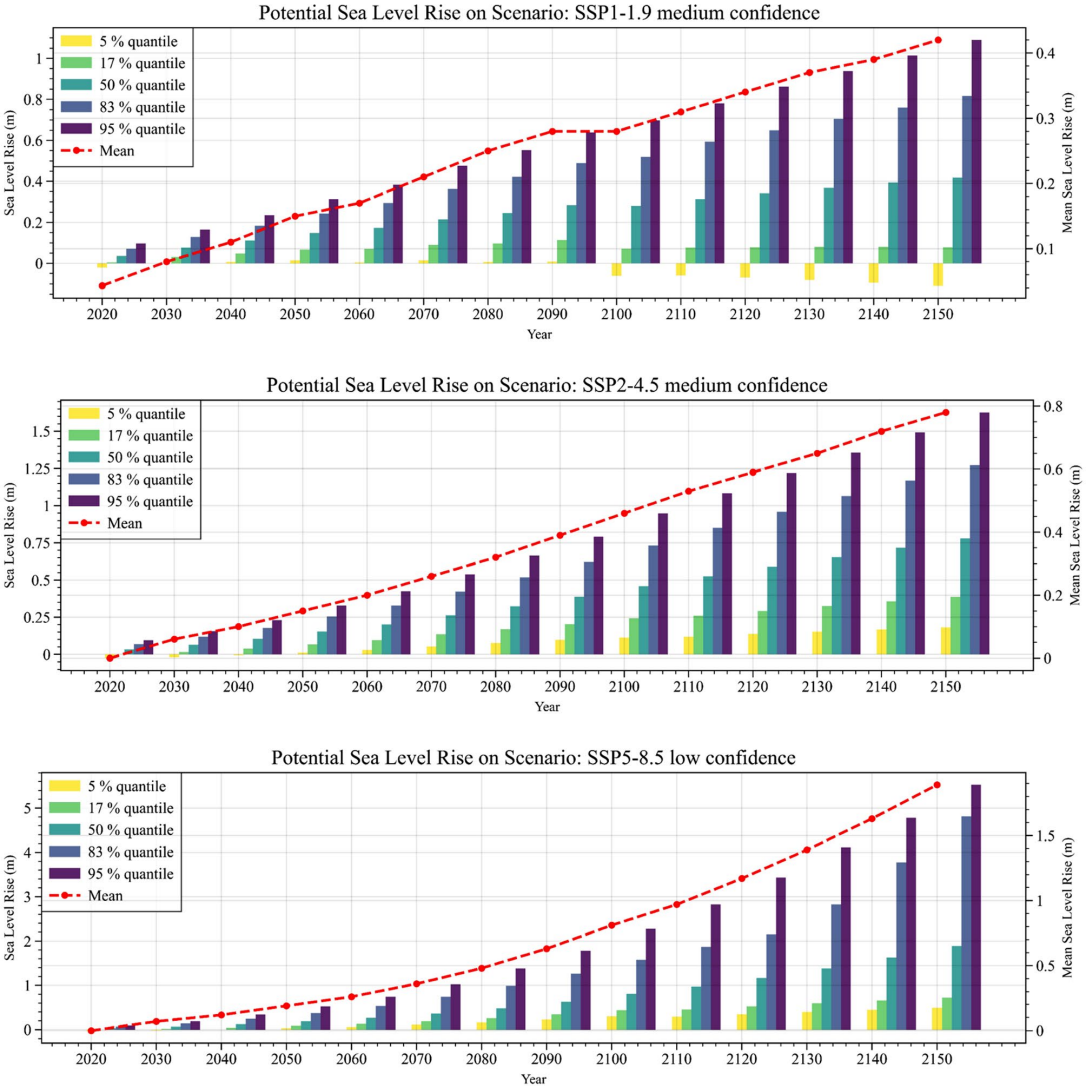
Potential SLR under selected SSP scenarios^5,35^ as per SSP1–1.9, SSP2–4.5, and SSP5–8.5. Each quantile shows the probability of the worst-case scenario and an SLR forecast based on emissions from 1995 to 2014.

From the IPCC’s AR6, two medium-confidence SSP scenarios (SSP1–1.9 and SSP2–4.5) and one low-confidence SSP scenario (SSP5–8.5) are selected for this study. The projected SLR trend was investigated based on the mean and several quantiles (5%, 17%, 50%, 83%, and 95%^7^) with an interval of 10 years between 2020 and 2150 (Fig. 1). Here, each quantile represents the percentage likelihood of the worst possible scenario and an SLR projection based on the respective scenario in comparison to average emissions from 1995 to 2014.

The mean sea level drops periodically in SSP1–1.9 and SSP2–4.5 (from 2020 to 2040), with a 5% quantile showing a positive impact on the earth (Fig. 1). Other quantiles for different SSPs show substantial sea level increases with temporal variation. The mean SLR according to SSP1–1.9 shows a yearly increase rate of − 0.84 mm/year to 8.38 mm/year from the 5 to 95% quantiles; slight increases from 1.4 to 12.516 mm/year occur under SSP2–4.5. The likelihood of such an SLR at this rate is comparatively high, as they present medium confidence levels. The mean SLR in SSP1–1.9 is projected to be 0.48 m by 2150. However, SSP5–8.5 shows a SLR of 3.78–42.507 mm/year according to the quantile variation, which is much higher than either of the previous two scenarios. Moreover, this scenario shows that the projected mean SLR can be 1.89 (0.72–4.81) m by 2150, compared to only 0.78 (0.39–1.27) m for SSP2–4.5. Overall, SSP5–8.5 shows a very high magnitude of SLR relative to other scenarios for the representative point.

### Spatiotemporal pattern of flood inundation in the region

**Figure 2:**
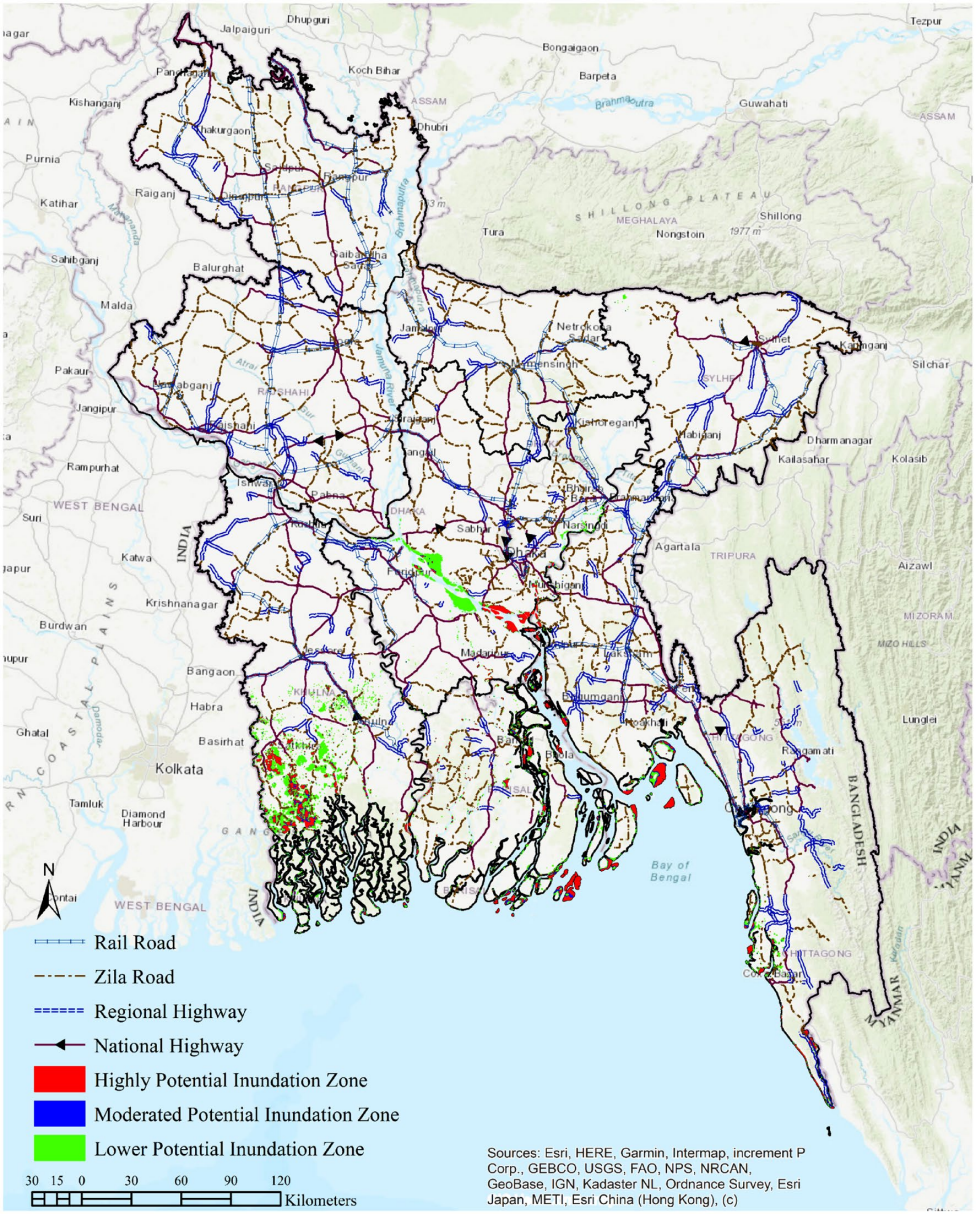
An inundation simulation to project the potential vulnerability of growing economic clusters in Bangladesh by 2150. A geographic information system (GIS) software package, ArcMap (Version 10.8, downloaded from https://bit.ly/45VbrPH), was used to generate the figure.

**Figure 3:**
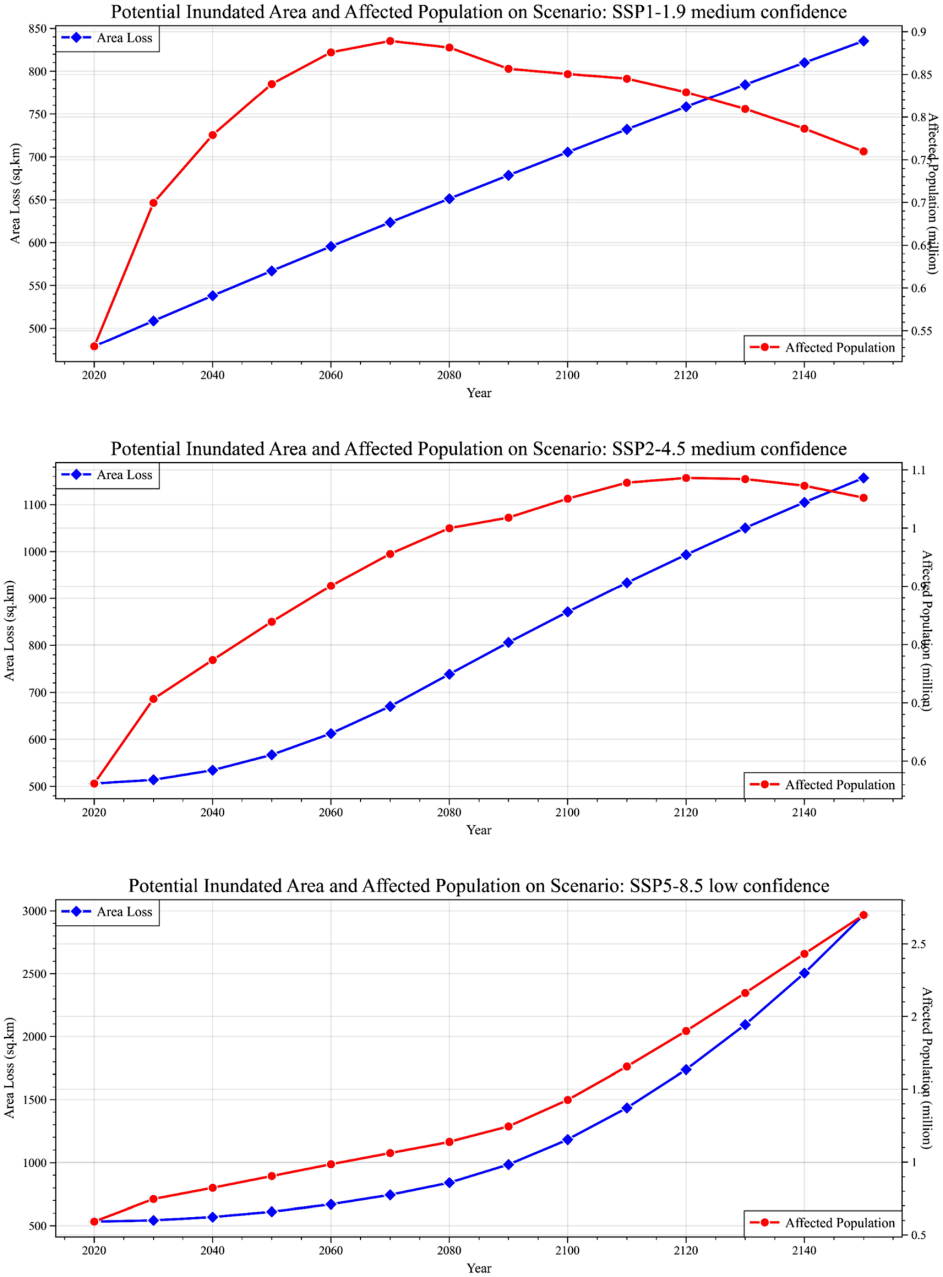
Potential inundation and affected population on temporal variation based on SSP scenarios. The calculation of the potential inundated area was based on the pixel count of the digital elevation model (DEM) with elevation values lower than the anticipated rise in sea level for each shared socioeconomic pathway (SSP) scenario.

Bangladesh is one of the largest coastal floodplains worldwide; consequently, low-lying parts of the country are more likely to be affected by a very small rise in sea level. Therefore, to demonstrate the possibilities of inundation, a GIS-based bathtub model was used based on the selected SSP scenarios. From the simulation, the projected inundated land area by 2150 can be observed (Fig. 3); the spatial distribution of this potential sea inundation is depicted separately (Fig. 2). Based on present global socioeconomic trends, the SSP1–1.9 scenario was considered the highest-possibility scenario, and the SSP5–8.5 scenario was considered the lowest-possibility scenario in this SLR projection. Figure 2 also represents the significant transportation routes throughout the country, which indicate higher population concentrations in urban locations.

Figure 2 shows that a greater portion of the coastal area of the BOB and adjacent plains of the Meghna delta fan is marked as being at a very high risk of sea inundation because its elevation is less than 0.45 ± 0.49 m. This includes the greater Khulna, Bagerhat, Satkhira, Jessore, and Narail from the southwestern region; Borguna, Potuakhali, and Bhola from the southernmost region; and Cox’s Bazar and part of Chittagong from the southeastern coast of the BOB. A few fragmented clusters of Lakshmipur, Noakhali, and Sharitpur are also estimated to be affected by sea inundation by 2150 under this projection. Even though the northern part of Bangladesh comprises higher elevations, a cluster of flooded zones may be observed across the lower elevated floodplains near the Surma–Kushiyara–Meghna River system.

The capital city, Dhaka, is projected to be at a partly high and mostly moderate risk of flooding from SLR. This is because Dhaka is next to the Buriganga River, which stages the early stage of Meghna Delta. Faridpur and Rajbari districts are moderate, but the northern parts of Shariatpur, Madaripur, and the southern part of Munshiganj are expected to be flooded by 2150, with a lower level of confidence. Port-city Chittagong, Sitakund, and Sandwip also have moderate-to-low risk factors with an elevation of lower 1.89 ± 0.49 m (Fig. 1). In contrast, Kutubdia, Matarbari, and St. Martin islands are projected to be high-risk areas for sea inundation.

### Projected inundation and its impact on the population

As discussed in the previous section, the spatial projections of mean sea inundation under various SSP scenarios are presented (Fig. 3). The potential inundated area was calculated using the number of pixels in the DEM that had lower elevation values than the projected SLR for each SSP scenario. These estimations were further related to the UN’s population density projection for Bangladesh.

According to the SSP1–1.9 scenario, sea inundation will range to 567.0963 km^2^, affecting approximately 0.83 million people by 2050. This inundation is projected to expand further by 705.4767 km^2^ in the 2100s and 835.3926 km^2^ by 2150, affecting approximately 0.8–0.85 million Bangladeshi inhabitants. Comparatively, a slightly higher inundated area is featured for SSP2–4.5, which indicates a transition of inundation from 567.16 to 1156.7 km^2^ within the study period. The expected affected population due to such inundation ranges between 0.8 and 1.05 million for the following years. However, for both scenarios, a decline in the affected population can be observed, as SSP1–1.9 and SSP2–4.5 estimate a significant decline in population and anthropogenic emissions after 2080–2100.

In contrast, SSP5–8.5 illustrates a very high magnitude of sea inundation, as this scenario models a future with a very high population density and a high frequency of energy consumption and emissions. The scenario depicts that the inundated area will extend approximately 611.32 km^2^, affecting approximately 0.9 million people in the 2050s, which is roughly equal to the other two SSP scenarios. In the 2100s, the inundated area will increase to 1183.2 km^2^, affecting almost 1.2 million habitats, and is projected to extend to 2967.5 km^2^ by 2150, affecting more than 2.7 million habitats in Bangladesh due to an expected mean SLR of approximately 1.89 m.

### Economic activity simulation under different scenarios

**Figure 4:**
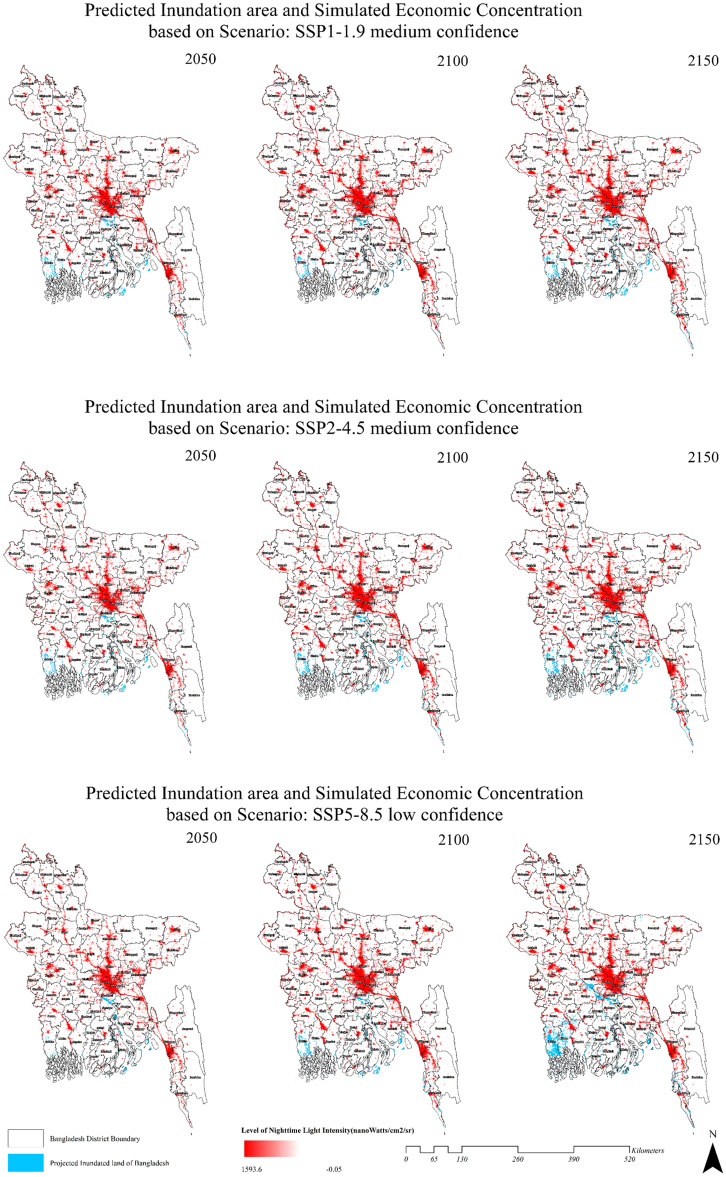
Spatiotemporal variation in sea inundation and its threat to potential economic clusters. The figure depicts the results obtained from the MOLUSCE simulation. The approach is evaluated through the utilization of specific SSP scenarios at 50-year intervals, with the aim of highlighting the potential consequences of sea inundation on Bangladesh's economic centers and urban settlements.

The MOLUSCE simulation plugin is used to determine the potential economic centers at risk due to sea inundation, as presented (Fig. 4). The method is assessed using selected SSP scenarios at 50-year intervals to emphasize the impact of potential sea inundation on Bangladesh's economic centers and urban settlements. The base years of 2014–2022 are chosen for economic and urban population concentration simulation, and an increase in variable clusters with temporal variation throughout all the districts of Bangladesh is evident. Major cities, such as Dhaka, Tongi, Gazipur, Rajshahi, Comilla, Chittagong, Sylhet, Pabna, Bogura, and Rangpur, have the highest potential for growth since they are important economic centers. Moreover, transition zones such as the Dhaka-Chittagong, Dhaka-Khulna, and Dhaka-Sylhet roads feature a significant increase in nighttime light radiance since they may function as rural‒urban fringe borders. A relatively high magnitude of nighttime light radiance may also be seen along the routes of Bogra-Rangpur, Bogra-Rajshahi, and Tangail-Rajshahi since the northern quarter of Bangladesh is expected to be significantly industrialized by 2030. Moreover, coastal cities such as Chittagong, Cox's Bazar, Khulna, and Mongla are anticipated to see growing demographic densities and commercial interest.

However, the potential economic and urban concentration zones have a high risk of sea inundation in different SSP scenarios. The sea inundation is heading from the southwestern coast to the southern coast, alongside the delta plains of the Meghna–Surma–Kushiyara River (Fig. 4). Significant sea inundation can also be observed along the floodplain of the Meghna–Padma stream in the western region and the Sangu–Matamuhuri–Karnaphuli river in the east for SSP5–8.5 starting in 2100. SSP1–1.9, although featuring a very low level of inundation, impacts several districts from the southern coast of Bangladesh. By 2150, rivers such as the Balaswar, Payra, Tetulia, and Arpangasia may become gateways for potential sea inundation and land loss in their adjacent major economic centers.

While SSP2–4.5 shows a frequent similar inundation pattern, with time, SLR may potentially move toward the districts of Khulna, Satkhira, Bhola, and Barisal, where economic concentration is expected to increase by 2100. Moreover, in this scenario, a few clusters in Chittagong and Cox's Bazar, as well as a significant percentage of Dhaka Division, are predicted to be vulnerable to sea inundation. Although the SSP5–8.5 scenario is expected to have lower confidence, major land areas where economic concentrations are much higher are projected to be inundated. This projection covers a large portion of the Padma-Meghna confluence and significant scattered clusters in the southern and southernmost parts of Bangladesh. The prediction places vast economic and urban centers, such as Chittagong, Khulna, Barisal, Mongla, and potentially sections of Dhaka and Sylhet, at risk. Remarkably, large plain areas adjacent to the Brahmaputra-Jamuna stream have a lower elevation profile (8 m), which may be critical to the inhabitants of Bangladesh's northern regions, such as Pabna, Kushtia, Rajbari, and Sirajganj. The key floodplains of Brahmanbaria, Kishoreganj, Habiganj, Netrokona, and Sunambaria could be inundated by the sea by 2150, according to the IPCC's worst-case scenario. This presents a concerning issue since the predicted visualization in Fig. 4 comprises 12.48% of the total land bodies, while SSP2–4.5 impacts 3.82% of the total land bodies in Bangladesh (Fig. 3).

## Discussion

Bangladesh is one of the fastest economically growing countries with a vast population. Therefore, changes in economic clusters are obvious^36,37^. Factors such as rural‒urban migration, rapid industrialization and urbanization, and geo-economic opportunity often cause changes in the economic orientations of the country^38,39^. As per our simulation (Fig. 4), a significant change in economic concentrations throughout the country, therefore, is evident. However, in the near future, the people of Bangladesh may need to move their settlements and economic centers due to climatic consequences such as SLR. From the above analysis, it is apparent that a significant portion of land area with socioeconomic prospects is at risk for sea inundation. Appropriate planning of the redistribution of the economic infrastructure may also be needed, as potential SLR may inundate and prevent prospectus zones from becoming inhabited. Considering the mean SLR, our study reveals that more than 0.5–2% of inhabited land may be submerged based on the present elevation profile, thus possibly evicting more than 2.8 million people by 2150^38,40^.

As per the IPCC’s SSP1–1.9 and SSP2–4.5 scenarios, the population density may be facing a degrading trend after 2080 along with a significant decline in GHG emissions. This may have a positive impact, lowering the ice sheet melting rate or seawater expansion (Fig. 2). However, the results from the worse-case scenarios (considering the 17 to 95% quantile) may cause up to 42.507 mm/year of SLR^2,4,35^. Moreover, contemporary subsidence in Dhaka and Khulna significantly reduces the elevation of land areas under the SLR threshold. A subsidence of 2.08 mm/year in Central Dhaka^17^ and 2.83 mm/year in Sundarbans^41^, Khulna, was measured. Furthermore, Rahman et al.^42^ reported a potential 170 km^2^ area loss in the Sundarbans between 1973 and 2010 due to subsidence, which is extremely concerning for the coastal belt ecosystem in Bangladesh.

The SLR in the BOB may also have a significant effect on the hydrology and morphological characteristics of the Meghna River, as the BOB receives a large amount of discharge from it. As the sea level rises, the rivers mentioned in preceding sections may need a wider catchment during the rainy season for additional discharges to prevent tremendous flooding and long-term rainwater logging. Higher chances of flooding the tributaries and distributaries are also expected and may cause significant inundation to nearby plains, as SSP5–8.5 denotes that the Padma River and upper Meghna River would have higher water levels. CEGIS, Dhaka, previously identified 40 new char areas (acquired lands) totaling approximately 1643 km^2^ in the Meghna estuary alone that have been developed during the previous 15 years^43^. Based on our study, these areas can be considered the most vulnerable areas even in the IPCC medium confidence scenarios. Our predictions also indicate that certain clusters of the districts of Kushtia, Meherpur, Pabna, Faridpur, Chandpur, Munshiganj, Narayanganj, Brahmanbaria, and Kishoreganj would face severe floods in their respective economic regions as a result of SLR. These districts comprise the crop hubs of Bangladesh.

The area in southern Bangladesh will be most affected by SLR in 2150, according to SSP5–8.5. By 2150, a significant part of Bangladesh's southern area is expected to be submerged under seawater. As a result, Bangladesh will lose several important economic areas, including Jessore, Faridpur, Madaripur, Chuadanga, Jhenaidah, Bagerhat, Gopalganj, Narail, Barishal, Chittagong City, and Cox's Bazar. The world’s largest mangrove, the Sundarbans, may also face a massive catastrophe since a significant number of land bodies are expected to be submerged under seawater while their adjacent distributaries rise. The inundation of Chittagong City would have a substantial adverse impact on Bangladesh's economy. The Port of Chittagong is used to convey more than 92% of imported and exported cargo. This is why the Port of Chittagong is referred to as the "Gateway of Bangladesh." The existence of this port has led the City of Chittagong to become one of several significant commercial centers in Bangladesh.

## Conclusion

This work demonstrates the use of an integrated application of sea inundation projections by utilizing hydrodynamic bathtub modeling and spatiotemporal analysis to evaluate the potential for various SLR scenarios in the BOB. The bathtub model was developed in response to the lack of readily available, user-friendly tools for modeling transitory coastal flooding^44–46^. Elevation data retrieved from Google Earth and the IPCC's AR6 offer an additional advantage by enabling the evaluation of numerous SLR scenarios^35^. Furthermore, our study utilizes an ANN-based population proxy nighttime simulation to include the trends underlying climate change scenarios, making it easier to identify future susceptible locations^33^. The findings of this paper indicate that considerable coastal (Khulna, Chittagong, and Cox’s Bazar) and low-lying floodplain (parts of Dhaka and Sylhet) areas are particularly vulnerable to potential SLR. These cities may be forced to endure significant financial and demographic consequences if severe SLR scenarios occur simultaneously. According to our projections, up to 2% of habitable land might be submerged, resulting in the relocation of approximately 2.8 million individuals by 2150. This submerged land includes the potential economic clusters of Bangladesh. Furthermore, possible changes in salt intrusion, habitat destruction, and ecological imbalance in the world’s largest mangrove^2,4,35^ can be identified from our spatiotemporal analysis.

However, we are aware that the extent of future climatic consequences is still uncertain in terms of future humanitarian responses. Administrations at all levels must incorporate the implications of climate change in their planning. The decision-support frameworks that rely on this research can be utilized in Bangladesh's coastal zone and lower-elevation floodplain planning. However, our study lacks the potential for practical planning to eradicate the consequences of SLR. Therefore, we recommend that future researchers focus on planning preventive mechanism to mitigate SLR consequences with a concentration on economic clusters. More precise topographic data, such as higher-resolution DEM data and valid statistics regarding mean subsidence in the region, should also be utilized in future studies to create more credible conclusions. Furthermore, any land-use changes should be accounted for based on local extreme weather occurrences.

## Materials and methods

### Study area

**Figure 5:**
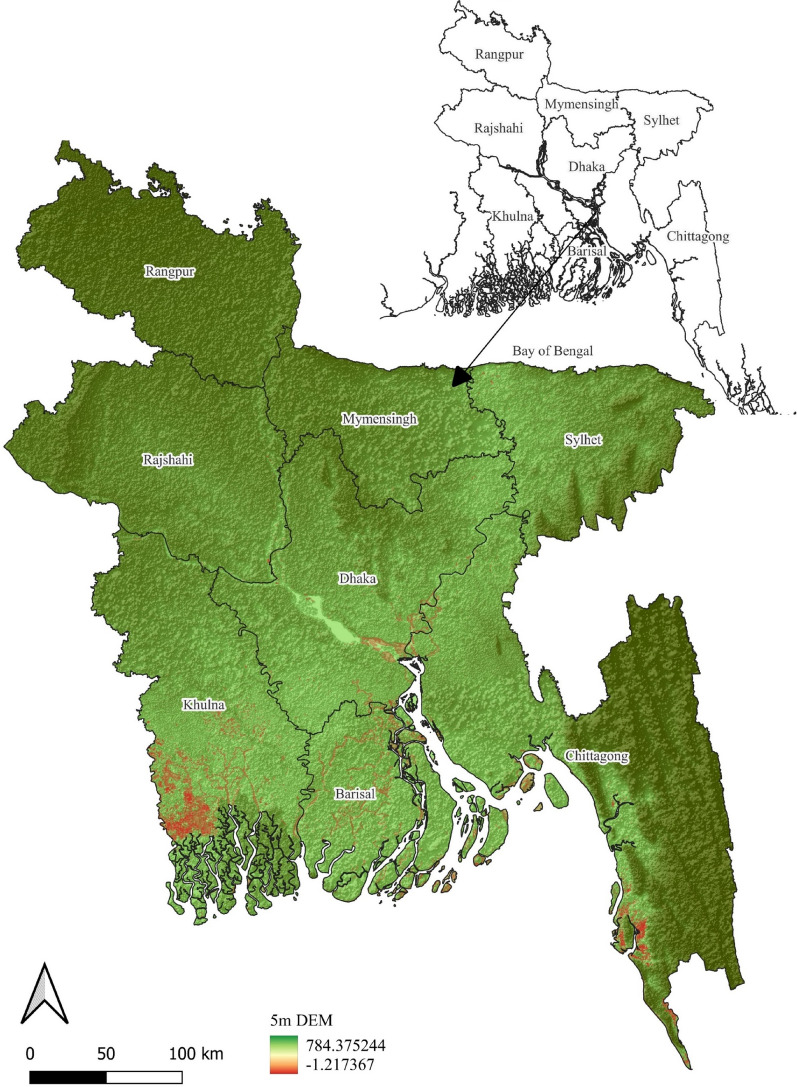
Elevation profile of Bangladesh, assessed with the SRTM digital elevation model. The GIS software packages ArcMap (Version 10.8, downloaded from https://bit.ly/45VbrPH) and QGIS (Version 2.18, downloaded from https://bit.ly/3qCUv0g) were used to generate the figure.

Bangladesh has a diversified landscape with a population of approximately 169 million people dispersed throughout 148,460 km^2^. The climate is a tropical monsoonal one, with hot, humid summers and moderate winters. The economy depends primarily on agriculture, including significant crops such as rice, jute, tea, and textiles. Its geomorphology is formed by the country's dynamic river systems, coastal activities, and tectonic activity. Hills and mountains are concentrated in the northern and eastern regions, while the Gangetic-Brahmaputra floodplain, one of the world's largest river deltas, dominates the central area. A low-lying deltaic plain caused by the confluence of multiple rivers forms the coastal area^47^. The Ganges–Brahmaputra Delta spans the western sector of the Bay of Bengal, formed by the sedimentation of the Ganges, Brahmaputra, and Meghna rivers (Fig. 5). Furthermore, in addition to the ongoing erosion and shifting caused by the dynamic interplay of river, tidal, and wave processes, the bay receives a large amount of deposition from Bangladeshi rivers. The bay is influenced by monsoonal rainfall, with significant rainfall during the southwest monsoon from June to September and the northeast monsoon from November to February^47^.

### Data

The NASA “Sea Level Projection Tool” data from the IPCC AR6 were used to evaluate the future SLR projection in the BOB, considering Cox’s Bazar point as the base^7^. This tool enables users to assess sea level projection statistics corresponding to the IPCC AR6^48^ and improves access and visualization of the report's consensus projections. It shows both regional and global sea level projections from 2020 to 2150 relative to a 1995–2014 baseline and how they differ considerably by scenario^5,35,49^. Sea level estimation utilizes uncertainty in emissions model temperature shifts as well as unpredictability in temperature-driver interactions, such as temperature expansion, ocean movement, and glacier and ice sheet melting. Here, the average rates of cumulative sea-level change are represented in mm year^−1^. IPCC scenarios are statistical techniques used to examine the parameter space of low- and high-emissions^50^. There is also information about how different physical processes will contribute to future SLR, which suggests which processes will be major contributors. In the dataset, quantiles spanning from the 17th to the 83rd are often interpreted as likely intervals, with "probable" meaning a likelihood of at least 66%. Thus, in this study, mean SLR data were used to project inundation in potential economic clusters by using SSP1–1.9 as a low emission scenario with a higher likelihood of uncertainty, SSP2–4.5 as a medium emission scenario with a moderate likelihood of uncertainty, and SSP5–8.5 as a high emission scenario with a low likelihood of uncertainty. Moreover, SLR under different quantiles was also analyzed to understand the magnitude of area loss based on the selected SSP scenarios.

The UN’s population projection data from IPCC AR6 were used to assess the affected population due to sea inundation^48,51^. The UN analyzes assessments of all countries' populations by age and gender once every 2 years in a report called World Population Prospects (WPP)^51–53^. The UN projections are developed based on multiple factors, e.g., future fertility, mortality, and global migration rates. Thus, the UN generates the "medium" prediction, as it is a single value for each prospective population^53^. Furthermore, the UN's population projections use the conventional cohort-component approach^54,55^.

Digital elevation models (DEMs) are often used in water resource studies to associate drainage attributes such as ridges, basin bottoms, channel networks, and surface hydrology with sub-floodplain channel size, length, and slope^56–60^. At present, multiple remotely sensed DEM datasets are available, e.g., NASA Shuttle Radar Topographic Mission (STRM), Thermal Emission and Reflection Radiometer (ASTER), Global Digital Elevation Model (GDEM), and European Space Agency (ESA) DEM over resolutions ranging from 30 to 900 m. However, this dataset may not be ideal for float-scale computations for precise hydrological research. Therefore, a 5 m resolution DEM of Bangladesh was generated for this study by assessing the elevation values in Google Earth Pro. Google Earth Pro is an enhanced version of Google Earth that enables the simultaneous representation of several locations as well as access to the elevation profile. Although Google Earth elevation data use SRTM as its elevation base data, several studies have confirmed its higher accuracy of elevation profiles compared to other available DEM datasets^61–63^. Moreover, the accuracy of a DEM improves with the level of denseness and unpredictability of the dots. In this study, the elevation data from Google Earth were extracted using Keyhole Markup Language (KML), with a total of 2.5 million elevation points covering the study region. The assessed point was further used to create a high-resolution (5 m DEM) raster dataset via inverse distance weighted (IDW) interpolation in ArcMap 10.8. Esri's ArcMap software is a complete platform for producing, collecting, analyzing, and demonstrating geographical data, making it popular among GIS professionals and scholars. In this study, ArcMap version 10.8 (downloaded from https://bit.ly/45VbrPH) was used. IDW specifically assumes that near objects are more similar than remote objects. IDW utilizes metrics around the prediction region to forecast any unrecorded value. Afterward, the modeled elevation profile was projected using the UTM WGS84 grid coordinate system in ArcMap. The resulting elevation map was then visualized in Fig. 5 via QGIS 2.18 to portray the spatial location of Bangladesh and the BOB. Quantum Geographic Information System, or QGIS, is popular open-source software that lets users produce, analyze, and display geographical data. The model used in this study is QGIS 2.18 (downloaded from https://bit.ly/3qCUv0g), which was selected in accordance with the study's computability.

NPP-VIIRS data were derived as a proxy for determining economic clusters. Numerous researchers employ it to estimate its economic effects on regional and national scales^64–66^. The Earth Observations Group (EOG) at the National Centers for Environmental Information (NCEI) produces monthly mean NPP-VIIRS images. This instrument has a resolution of 15 arc seconds. The VIIRS sensor aboard the Suomi National Polar-Orbiting Partnership (NPP) satellite analyzes data in 22 distinct wavelength bands, one of which is the DNB (Day/Night Band). VIIRS DNB statistics have been used to evaluate the population, assess working conditions in remote modernization, monitor natural hazards and disharmony, and comprehend light pollution's biological impacts^32,64,66^. Mean radiance composite images are made with data from the VIIRS Day/Night Band based on nighttime brightness.

## Simulation models

### Change analysis using a cellular automata model

Satellite-based nighttime light images have been broadly used by economists as a substitution to determine economic interaction in poor countries. Cities and towns with strong social and cultural infrastructures show greater population dynamics^64–66^. Furthermore, it is pertinent to forecast the potential consequences of SLR in these economic areas. Therefore, to evaluate spatiotemporal changes and predict the future economic changes between the study years, the MOLUSCE plugin on QGIS was used. The MOLUSCE plugin makes use of cellular automata data to simulate hypothetical outcomes based on past ones^67^. It evaluates altering analysis and transformation prospects via four methods: logistic regression, weights of evidence, multicriteria evaluation, and artificial neural network (multilayer perceptron). Such approaches construct a parameter that forms the foundation for the prediction model^34,68^. In this study, several nighttime DNB clusters from 2014 and 2020 were applied with the QGIS MOLUSCE tool to generate a transition matrix. The MP-ANN approach in QGIS 2.18 software is used to train a model of preceding nighttime light transitions^69,70^. The plugin was used to forecast the scenarios for 2050, 2100, and 2150, utilizing nighttime light visuals as a spatial parameter.

1 $${\text{LU}}_{{{\text{t}} + 1}} = \, {\arg\max}_{\text{j}} \left\{ {{\text{P }}\left( {{\text{LU}}_{{{\text{t}} + 1}} = {\text{ j }}|{\text{ LU}}_{{\text{t}}} = {\text{ i}}} \right) \, *{\text{ LUP}}_{{{\text{t}}({\text{i,j}})}} } \right\}$$

The abovementioned Eq. (1) demonstrates the logistic regression that is used to detect the potential changes in the training raster dataset. Here, LU_t+1_ = land-use class at time t + 1, argmax_j_ = class that maximizes the product of transition likelihood and current land-use pattern, P (LU_t+1_ = j | LU_t_ = i) = transition probability from land-use class i to j at time t + 1, and LUP_t(i,j)_ = current land-use pattern value for the transition from land-use class i to j at time t.

Furthermore, the MP-ANN model was validated by contrasting the simulated and estimated values for 2018, 2021, and 2022 DNB composites using MOLUSCE QGIS validation. It is a nonlinear statistical analysis approach that prepares urban development drivers and accounts for intricate underlying characteristics during modeling^71^. The statistical alterations of an ANN have generally weighted sums of materials, activation coefficients, and bias variables, and their formulae are dependent on the design of the neural network (e.g., feedforward, recurrent, convolutional) as well as the modeler's stimulation functions and other factors. MOLUSCE's ANN is coupled with the cellular-automata (CA) simulation approach. CA employs the Monte Carlo algorithm technique, which is thought to be compatible with predicting land-use change^72^.

2 $$Y = \frac{1}{N}\left( {\sum {f(x_{i} )} } \right)$$

In Eq. (2), the Monte Carlo simulation creates independent samples from probabilistic distributions that represent the system's unanticipated variables or characteristics. Here, *Y* = estimated outcome, *N* = number of samples, *f*(*x*_*i*_) = value of the function at the ith random sample, *x*_*i*._

**Figure 6:**
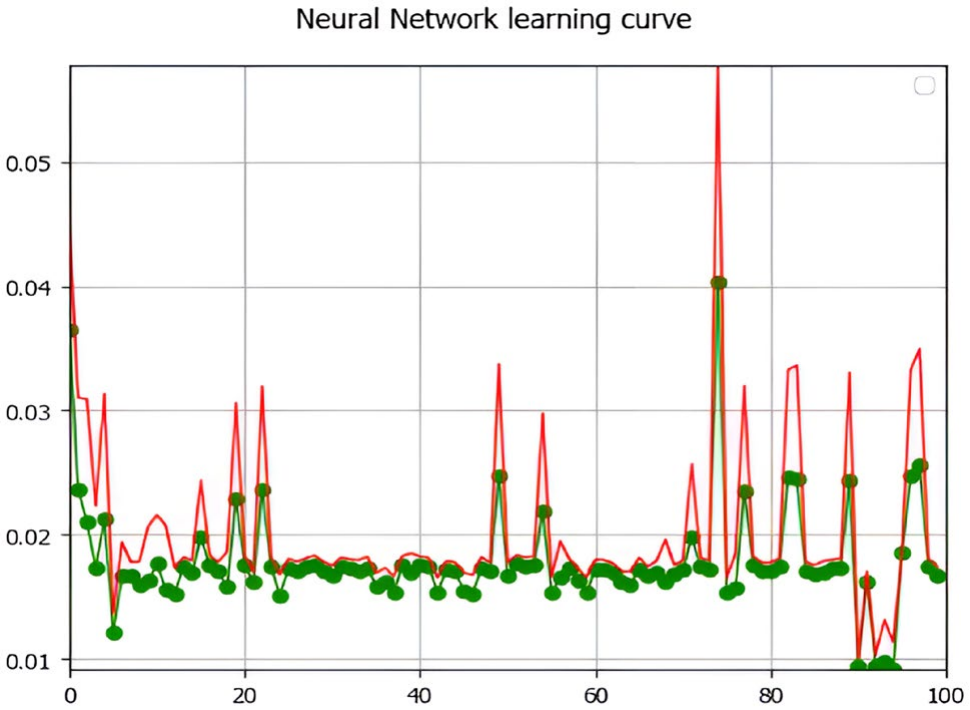
Neural network learning curve of the DNB composite images of the potential nighttime light clusters to project future economic clusters (the red line represents the training set performance, whereas the green line represents the validation set performance).

Furthermore, to eliminate dataset inconsistencies, a raster calculator was used to scale all the raster images between 1 and 5 with similar intervals before running the model. The neural network learning curve with a maximum iteration of 2000 is presented in Fig. 6.

Figure 6 displays the network's efficacy on the training and test datasets as an accumulation of training epochs from the training data periodically. The red line in this learning curve represents the training set performance, whereas the green line represents the validation set performance. Furthermore, the result of the training dataset will demonstrate the kappa validation value in Table 1. The overall kappa (*k*_*i*_) (Eq. 3) and % of correctness (C) (Eq. 4) were determined as follows:

3 $$k_{i} = \frac{{P_{0} - P_{e} }}{{1 - P_{e} }}$$

4 $$C = \frac{{\sum\nolimits_{i = 1}^{k} {n_{ij} } }}{n}$$

**Table 1:** Validation parameters (K parameters) and % correctness of the CA-ANN model in QGIS software.

Simulated year	2018	2021	2022
% Correctness	89.622	97.399	89.803
K histogram	0.713	0.920	0.729
K location	0.806	0.839	0.913
Overall kappa	0.695	0.772	0.798

Here, *P*_*0*_ = observed proportion of agreement, *P*_*e*_ = proportion expected by chance, *n*_*ij*_ = diagonal elements in the error matrix, *k* = total number of classes, and *n* = total number of samples in the error matrix.

However, the DNB composite simulation accuracy was evaluated using a percent correctness value that exceeded > 85% (Table 1).

### Bathtub approach

The bathtub model, or bathtub approach, is a frequently used framework for assessing flooding worldwide^60^. It is a geospatial technique that simulates coastal surface runoff via digital elevation models and is dependent on the accuracy of the geomorphologic input data^73^. A bathtub model considers potentially vulnerable regions that might be below the inundation level and hydrologically associated with the source of flooding (e.g., the ocean or river). Therefore, several researchers have implemented this approach to monitor the flooding prospects of coastal areas or, in conjunction with socioeconomic and infrastructure data, to assess the flood inundation risk^74,75^. The bathtub methodology can be performed in GIS software, which allows for easy incorporation with other geodatabases or even in matrix computational development tools^73^.

However, for the current study in which certain SSP scenarios and years were chosen, the bathtub approach was applied to evaluate areas adjacent to the BOB that could potentially be inundated due to SLR. The predicted submerged land bodies of Bangladesh due to sea inundation (Figs. 2 and 4) in IPCC AR6^35^ were analyzed using ArcMap 10.8. The spatial distribution of potential sea inundation is visualized in Fig. 2 by using the same ArcMap version to highlight the potential hazard of substantial manmade buildups in Bangladesh. The publicly-available shapefiles of rail and road networks were collected from https://geodash.gov.bd/. The elevation profile of distinct IPCC-proposed hazard scenarios was used to develop nine raster files for the evolution of SLR scenarios in Fig. 4. For further analysis, reclassified rasters were transformed into polygons.

## Data Availability

The data that support the findings of this study are available from the corresponding author, Muhammad Muhitur Rahman (mrahman@kfu.edu.sa), or the first author, Bijoy Mitra (bijoymitra11@gmail.com), upon reasonable request.
